# A Systematic Review of Empirical Evidence on Art Therapy With Traumatized Refugee Children and Youth

**DOI:** 10.3389/fpsyg.2022.811515

**Published:** 2022-05-18

**Authors:** Nadia Annous, Anies Al-Hroub, Farah El Zein

**Affiliations:** ^1^American University of Beirut, Beirut, Lebanon; ^2^Emirates College for Advanced Education, Abu Dhabi, United Arab Emirates

**Keywords:** art therapy, trauma, refugees, post-traumatic stress disorder, systematic review, evidence-based practice, quality indicators, mental health

## Abstract

The current global refugee crisis revealed that refugee children, youth, and adults are uniquely vulnerable to traumatic events. Yet, there are only a few studies available that report robust systematic data on art therapy interventions with mental health in recent refugee populations. The purpose of the study is to synthesize and evaluate (a) the available research evidence on the use of art therapy in reducing post-traumatic stress disorder (PTSD) levels in refugees, and (b) the quality of empirical evidence for each of the reviewed studies. The authors adopted the Council for Exceptional Children (CEC) evidence-based practice (EBP) standards and quality indicators to evaluate the methodological soundness of the reviewed studies and the evidence-based classification of art therapy as a treatment intervention. We systematically searched electronic databases of pertinent review articles for the period from 2010 to 2020 using the Preferred Reporting Items for Systematic Reviews and Meta-Analyses (PRISMA). Systematic searches identified 70 research articles but yielded eight eligible journals as per the inclusion criteria. Results indicated that, though considered a promising treatment approach, art therapy is presently classified as an intervention that falls under the category of practice with insufficient evidence. The findings suggest the need for further methodologically sound experimental studies to strengthen the evidence behind art therapy as an intervention to reduce PTSD symptoms in refugees around the world.

## Introduction

Post-traumatic stress disorder (PTSD) is one of the common psychological and anxiety disorders that are present among refugees (Khamis, 2019; Grasser et al., 2021). PTSD is characterized by severe symptoms of re-experiencing and avoidance due to traumatizing experiences and thus leading to impairment in important areas of functioning such as social areas, occupational areas, and other crucial areas (American Psychiatric Association, 2013). Meta-analyses conducted by Lindert et al. (2017), Blackmore et al. (2022), and Henkelmann et al. (2020) showed that refugees have the highest PTSD prevalence rates, 32, 31.46, and 29% respectively. Systematic review research showed that refugees suffering from PTSD exhibited severe symptoms, such as intrusion, withdrawal, negative mood, alternations in arousal (Ghumman et al., 2016), hypervigilance, difficulties falling asleep, irritability, outbursts of anger, self-destructive, reckless behavior (Spiller et al., 2017), anxiety, depression, and somatization (Lindert et al., 2017; Nesterko et al., 2020; Verhülsdonk et al., 2021). Moreover, when children are exposed to refugee conditions, the symptoms of their trauma might begin later in adolescence (Cohen et al., 2006; Al-Hroub, 2014; Saab et al., 2019; Al-Hroub et al., 2020). In a systematic review conducted by Kien et al. (2019) on mental disorders in young refugees and asylum seekers in European countries, findings showed that the prevalence varied widely among 47 studies covered in 53 articles for the period from 1990 to 2017 for PTSD (19.0% and 52.7%), depression (10.3 and 32.8%), anxiety disorders (8.7% and 31.6%), and emotional and behavioral problems (19.8% and 35.0%).

Therefore, the role of psychosocial, therapeutic, and mental health interventions is found crucial in managing PTSD symptoms in refugee children and youth (Giacaman et al., 2007; Al-Hroub, 2015; Almoshmosh, 2016; McLaughlin and Al-Hroub, 2016), and adults (Palic and Elklit, 2011). Research showed that therapeutic practices that rely on non-verbal treatment, such as art therapy, may promote healing refugee learners affected by war (Harris, 2009; Rowe et al., 2017; Zubala and Karkou, 2018).

### The Role of Art Therapy

Art therapy is known as an interdisciplinary field where art therapist combines approaches from different fields such as art education, counseling, neuroscience, visual art, and others (Bucciarelli, 2016). Art therapy has been employed as an intervention to reduce PTSD symptoms since it provides a non-threatening environment for refugee children and adolescents to facilitate the expression of feelings that are linked to trauma and identify some feelings that can lead to a sense of relief. Art therapy also provided the opportunity for refugee learners to tolerate their negative emotions when they become capable of regulating their emotions (Sommers-Flanagan, 2007; Case and Dalley, 2014; Kalmanowitz and Ho, 2016; Akthar and Lovell, 2019; Wahlbeck et al., 2020). In addition, refugee learners who have difficulties in verbal expression find art therapy very effective (Stuckey and Nobel, 2010).

Meta-analysis research has shown that the use of artwork applied by therapists and practitioners aids not only learners who have been diagnosed with PTSD (Schouten et al., 2015), but also those with autism spectrum disorder (Schweizer et al., 2014), depression (Blomdahl et al., 2013), anxiety (Abbing et al., 2018), and other mental illnesses (Kaye-Huntington and Peterson, 2010). However, research results for policy decision-making and implementation processes regarding therapeutic procedures in art therapy are still limited (Damianakis, 2007; Register and Hilliard, 2008; Schouten et al., 2015).

In practice, art therapy involves the product, the process, and the relationship between the counselee and the therapist. However, the diversity of approaches in art therapy due to several factors is reflected in the emergence of different titles and different approaches such as person-centered art therapists, group analytic art therapists, cognitive, gestalt, cognitive-behavioral art therapy, and studio approaches to art therapy (Edwards, 2014). Although each approach has its theory and techniques, the use of art as the language of expression is what they have all in common.

The classification of art therapy distinguishes between three approaches: directive, non-directive, and combined. In a directive approach, the session usually starts with the therapist introducing themes to facilitate the creative activity. After an engagement in the creative activity, the session would end with a discussion as members in the group discuss, share thoughts and feelings, and share their work (Edwards, 2014). In the non-directive approach, the therapist avoids giving directives or themes believing that imposed themes or structures may inhibit the natural discovery within the person (McNeilly, 1983). Art, language, and literature from psychology can be combined in various ways to better affect positive change, and art by itself can be considered a language of expression (Morrell, 2011). Regardless of the adopted approach, learners who are referred for art therapy do not necessarily have to be skillful in art as the aim is to offer the opportunity to express and not assess the art produced (Case and Dalley, 2014).

The rationale of this research stems from the drastic increase of forcibly displaced refugees in the world and the increasing need for providing psychosocial non-verbal interventions to deal with their traumas globally. According to the United Nations High Commissioner for Refugees (UNHCR), more than 84 million people were forcibly displaced worldwide by the first half of 2021 as a result of persecution, conflict, violence, human rights violations, and events, of which 26.6 million have been acknowledged as refugees (United Nations High Commissioner for Refugees, 2021). Over the period between 2010 and 2020, the population of refugees increased ~3.6 times [from 7.2 million (United Nations High Commissioner for Refugees, 2010) to 26 million]. In parallel, there has been increasing attention in research in studying the effectiveness of alternative and creative art therapy on the social and emotional, and health wellbeing of learners since the beginning of the twentyfirst century, particularly in the past decade (Clift et al., 2019). Therefore, this study explores, through a systematic review of empirical research, the use of art therapy in reducing PTSD levels in refugee children from 2010 to 2020.

Several systematic review studies have explored the effectiveness and contribution of art to children with clinical conditions, such as trauma, special education and disabilities, non-specific difficulties, medical conditions, and juvenile offenders (e.g., Cohen-Yatziv and Regev, 2019), children, and adolescents with psychosocial problems (Bosgraaf et al., 2020), or traumatized adults (Schouten et al., 2015), and adults with depression, borderline personality disorder, schizophrenia, and PTSD (e.g., Van Lith, 2016). Yet, none of these studies explored the effectiveness of art therapy on refugee children and adolescents with PTSD.

Thus, the present study aims to synthesize and evaluate the available research evidence on the use of art therapy in reducing PTSD levels in refugees over the years between the years 2010 and 2020. In addition, the study aims to evaluate the quality of empirical evidence for each of the reviewed studies by adopting highly regarded and reliable quantitative indicators. Given the research aims, a systematic review of the literature was warranted to address the following research questions:

a. What is the available research evidence on the use of art therapy in treating PTSD traumatic symptoms in refugees between the years 2010 and 2020?
b. What are the qualities of empirical evidence for available peer-reviewed studies on art therapy practices with traumatized refugees?

## Method

### Research Design and Search Process

**Figure 1:**
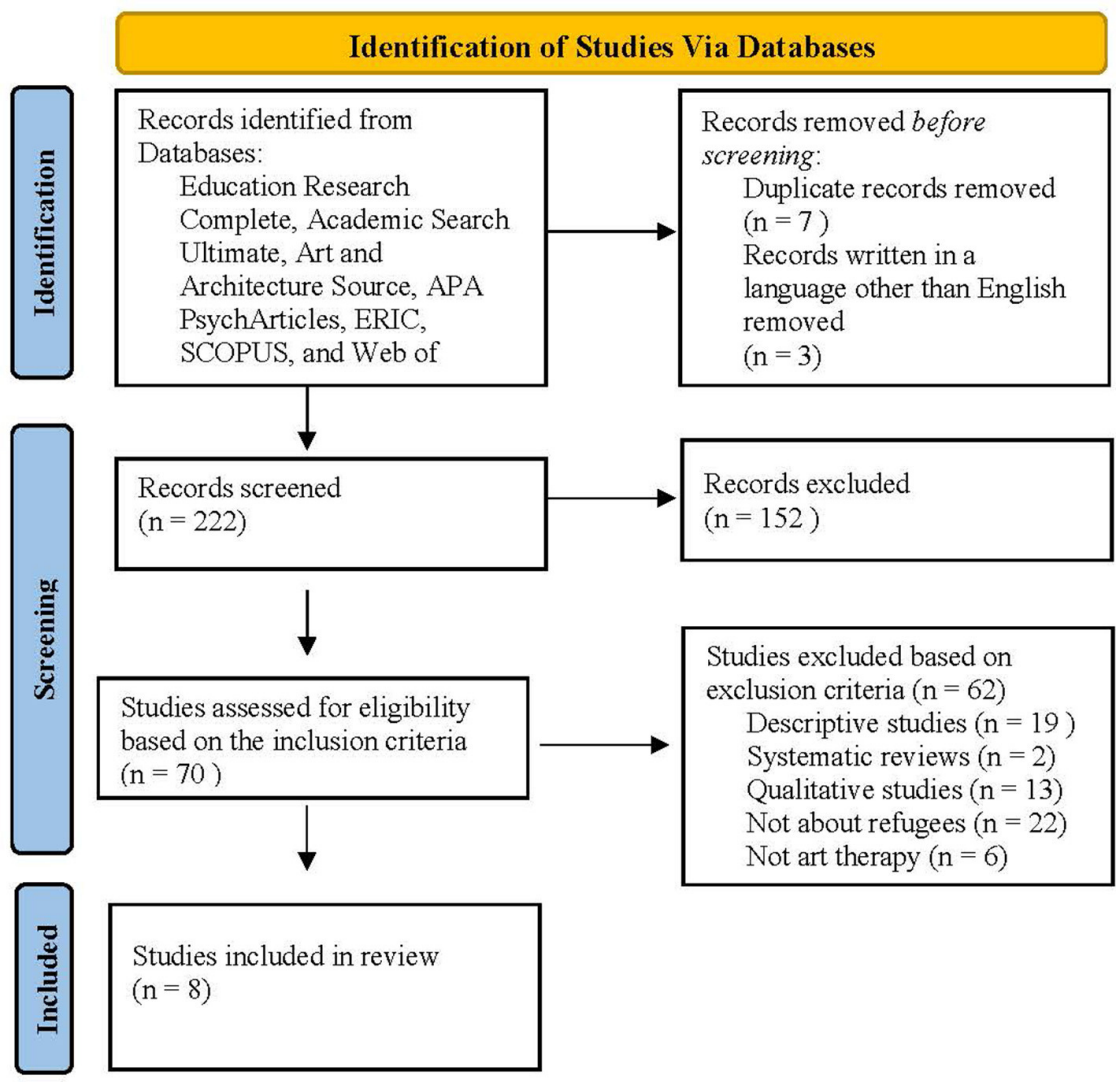
PRISMA flow diagram for systematic review. From: Page et al. (2021).

A systematic review of research related to the use of art therapy with traumatized refugee children, adolescents, and adults was conducted using Preferred Reporting Items for Systematic Reviews and Meta-Analyses (PRISMA; Page et al., 2021). Peer-reviewed studies were identified through the following Boolean search terms entered in Education Research Complete, Education Research Complete, Academic Search Ultimate, Art and Architecture Source, APA PsychArticles, Web of Science (WOS), Scopus, and ERIC databases: (“trauma,” OR “PTSD,” OR “post-traumatic stress disorder,” OR “traumatized children”) AND (“refugee^*^” OR “forced migration”) AND (“art therapy” OR “art”). The Boolean terms were entered into the mentioned databases and the search process continued across the three PRISMA phases (see Figure 1). The search was filtered to include articles published in English between the years 2010 and 2020. Two professors of special education reviewed the keywords and agreed on the list of terms that can be used during the search procedure. For relevance to the current review, the abstracts, as well as full-text peer-reviewed articles were obtained and screened. The systematic review focuses on experimental studies conducted from 2010 to 2020. Given that there are a limited number of studies on art therapy with refugee children, this topic was explored at a broader level with a focus on adults, adolescents, and children refugees.

### Applying the Inclusion and Exclusion Criteria

In the first phase, databases were selected to search for the selected articles related to the psychology field. The reports obtained were then screened by examining the title and abstract of each article. Results obtained from the screening phase were then examined more closely (i.e., reading full text) and the following inclusion criteria were applied:

(a) Peer-reviewed journal articles,
(b) Published during 2010-2020,
(c) Art therapy intervention study (group design or single-subject research design),
(d) All study sample sizes,
(e) Intervention conducted with refugees diagnosed with PTSD, and
(f) A study published in the English language.

Systematic reviews, descriptive articles, book reviews, and qualitative studies were excluded. Additionally, studies that investigated the effects of expressive art therapy (e.g., dance, music, drama) and other therapeutic interventions that do not include art were removed from the list for potential inclusion. Dissertation and thesis studies were also excluded.

The literature search was conducted, independently, by three researchers (N.A. A.A, and F.Z), who also independently screened the identified peer-reviewed articles' titles and abstracts to assess their eligibility as per the inclusion criteria. If it was unclear whether a study was eligible, the study article was assessed in full. The two researchers concluded that eight research articles yielded eligible.

### Coding Based on CEC Quality Indicators and Evidence-Based Classifications

The CEC quality indicators and evidence-based classifications were used to assess the certainty of the evidence for each of the reviewed studies (Cook et al., 2014). The content-coding table was divided into eight areas presenting the quality indicators: (a) context and setting; (b) participants; (c) intervention agent; (d) description of practice; implementation fidelity; (f) internal validity; (g) outcome measures/independent variable; and (h) data analysis. Percentages to determine the quality indicators met are calculated after coding the elements across the studies. After reading the article and identifying the elements in each article, the element was coded as 1 if there is sufficient information regarding the indicator element and 0 if there is insufficient information for the indicator element by referring to the coding sheet based on the CEC standards (Cook et al., 2014).

The CEC standards include 28 quality indicators. Eighteen of the quality indicators apply to group comparison and single-subject studies, six apply only to comparison studies and four are specific to single-subject studies (Cook et al., 2014). The study is expected to describe the critical features regarding the context and setting. To understand the population that will benefit from the intervention, it is important to describe clearly the participants' demographics and define clearly the difficulty of focus or disability the criteria for determining the disability or the difficulty. Researchers also need to examine the role of the intervention agent and background variables. The study is also expected to provide a detailed description of the intervention procedures and intervention agents' actions. The practice also has to demonstrate the fidelity of implementation by using direct reliable measures. For internal validity, the study is expected to describe the baseline and provide a clear description of the assignment to groups. Researchers should include detailed information on the comparison/control group and how participants are assigned to ensure the comparison conditions are meaningful. The researchers are expected to consider the outcome as an indicator by examining how the study clearly defines and describes the measurement of variables and how the effects are reported on all measures of the outcome. Moreover, the researchers need to show the appropriateness of frequency and timing of outcome measures. Finally, data analysis should be conducted appropriately by examining the data analysis techniques such as effect size calculations to determine the amount of the effect of an intervention on the participants or the group.

The quality indicator for each element was calculated individually to determine the percentage met for each indicator across each study. To ensure reliability, two researchers read and coded each of the 8 elements to confirm the identified results and the information. A point-by-point method was used to establish interrater reliability for coding articles. All articles were double-coded by an independent researcher, and the percentage of agreement was calculated (i.e., agreements divided by agreements plus disagreements, multiplied by 100). The initial percentage of agreement was 93%. Coding results were communicated, inconsistencies were discussed, and a consensus was reached (i.e., 100% agreement).

### Evidence-Based Classification

After assessing the reviewed studies for quality indicators, methodologically sound studies are then evaluated for evidence-based classifications. A study is considered for evidence-based classification only when they use group comparison designs (e.g., randomized or non-randomized quasi-experiments) or a single-subject research design (Cook et al., 2014). Eligible studies are classified as (a) evidence-based, (b) potentially evidence-based, (c) mixed-effects, (d) insufficient evidence, or (e) negative effects based on the number of single subjects, and group comparison studies of strong and moderate methodological quality with positive, neutral, and negative effects (Cook et al., 2014). *Evidence-based practices* (EBP) are supported by two group comparison studies with random assignment to groups and at least 60 participants across studies, four group comparison studies with the non-random assignment to groups, and at least 120 participants across studies, or five single-subject studies with at least 20 participants across studies. To be identified as EBP. The practice has to also meet 50 % of the criteria for two or more of the study designs. The group of studies on a given practice should not include any studies with negative effects and at least a 3:1 ratio of positive effects to neutral/mixed effects. *Potentially evidence-based practices* must be supported by one group comparison study with random assignment to groups and positive effects; two or three group comparison studies with no randomly assigned groups and positive effects; or two or four single-subject studies with positive effects. For this item, CEC considers group experimental, non-randomly assigned group comparison, and single-subject design studies collectively. *Mixed-evidence practices* must meet the criteria for evidence-based practice or potentially evidence-based practice and the ratio of studies with positive effects to studies with neutral/mixed-effects is <2:1 or one or more studies with negative effects if these studies do not outweigh studies with positive effects. *Insufficient evidence* exists when the literature is lacking all the other evidence-based criteria. *Negative effects* must include more than one sound study with negative effects, and the number of studies with negative effects outnumbers the sound studies with positive effects.

## Results

Initially, the Boolean search yielded two hundred thirty-two (*n* = 232) results, from which seven duplicated records and three records not written in the English language were removed. Two hundred twenty-two (*n* = 222) results were obtained before applying the inclusion criteria. After screening titles and abstracts, seventy (*n* = 70) articles were considered for potential inclusion. The full texts of the seventy peer-reviewed articles (*n* = 70) were read and examined. The application of the above-mentioned inclusion criteria yielded eight experimental studies for review (see Figure 1). The reviewed studies employed variations of group designs: quasi-experimental, pre-post group design without control, and within-group designs. Methodological soundness for each study was evaluated based on CEC quality indicators, and quantitative results are reported in Table 1 and explained below. Table 2 represents information about the intervention context, sample size, nationality, and age group of participants across reviewed studies. Participant age groups varied from 7 to 80 years, and the sample size ranged from 12 to 145 participants.

**Table 1:** Methodological soundness by quality indicators.

**Quality indicator**	**Moosa et al. (2017)**	**Rowe et al. (2017)**	**Feen-Calligan et al. (2020)**	**Schouten et al. (2019)**	**Ugurlu et al. (2016)**	**Meyer DeMott et al. (2017)**	**Van Wyk et al. (2012)**	**DroŽdek et al. (2014)**	**Quality indicator met %**
Context and setting	1/1	1/1	1/1	1/1	1/1	1/1	1/1	1/1	100%
Participants	2/2	2/2	2/2	2/2	2/2	2/2	2/2	2/2	100%
Intervention agent	1/2	2/2	2/2	1/2	2/2	2/2	2/2	1/2	81%
Description of practice	2/2	1/2	2/2	1/2	1/2	2/2	1/2	1/2	69%
Implementation fidelity	0/3	2/3	1/3	2/3	0/3	0/3	0/3	0/3	21%
Internal validity	2/6	2/6	3/6	3/6	2/6	4/6	3/6	2/6	44%
Dependent variables	5/6	5/6	5/6	5/6	5/6	5/6	5/6	6/6	85%
Data analysis	2/2	2/2	2/2	2/2	2/2	2/2	2/2	2/2	100%
Quality indicators met %	63%	71%	75%	71%	63%	75%	67%	63%	

**Table 2:** Context and demographic information across reviewed studies.

**Author(s)**	**Research design**	**Sample**	**Nationality**	**Context**	**Age by year**
Moosa et al. (2017)	Pretest posttest design without a control group	30	Sharam Vihar and Mehrath	India	14–18
Rowe et al. (2017)	Pretest posttest design without a control group	30	Karen, Burmese	Burma	11–20
Feen-Calligan et al. (2020)	Quasi-experimental	15	Syrian	U.S.A	7–14
Schouten et al. (2019)	Pretest posttest design without a control group	12	Russia, Iraq, Bosnia, Iran, Congo, Afghan, Ireland, Netherlands	Netherlands	18+
Ugurlu et al. (2016)	Pretest posttest design without a control group	63	Syrian	Turkey	7–12
Meyer DeMott et al. (2017)	Quasi-experimental	145	Afghan, Somalian	Norway	15–18
Van Wyk et al. (2012)	Pretest posttest design without a control group	62	Burmese	Australia	18–80
DroŽdek et al. (2014)	Pretest posttest design without a control group	69	Iranian and Afghan	Netherlands	18–70

The evidence so far supporting art therapy for treating PTSD in refugees includes: (a) two quasi-experimental design studies (Meyer DeMott et al., 2017; Feen-Calligan et al., 2020), (b) five pre-test post-test group studies with no control (Van Wyk et al., 2012; Ugurlu et al., 2016; Moosa et al., 2017; Rowe et al., 2017; Schouten et al., 2019); and (c) one within-subject group design with no control (DroŽdek et al., 2014). Systematic coding of each of the eight articles based on CEC quality indicators indicated that none of the reviewed studies received a score that is below 50% with overall scores ranging from 59 to 86%. Detailed results in relation to our evaluation of quality indicators per study and evidence-based classification of the overall treatment practice are illustrated and justified below.

### Results for Quality Indicators

Findings show that all reviewed studies met the criteria regarding describing the context and the setting explicitly. Studies took place in different locations such as Turkey, Sharam Vihar, Mehwath, Australia, and the United States. Similarly, all reviewed studies met the criteria related to providing sufficient information on participants' demographics. It is also critical to note that none of the studies that involved more than one group used random participant assignment to study groups (DroŽdek et al., 2014; Meyer DeMott et al., 2017; Feen-Calligan et al., 2020). The sample size across studies varied from 12 to 145 participants, and the age of participants ranged from seven to 80 years.

Five articles met the criteria for both intervention agent quality indicator elements (Van Wyk et al., 2012; Ugurlu et al., 2016; Meyer DeMott et al., 2017; Rowe et al., 2017; Feen-Calligan et al., 2020). In the studies conducted by Schouten et al. (2019) and by DroŽdek et al. (2014), the qualifications, certification, or description of the training of the art therapist were not mentioned. Similarly, the researcher's background or certification, or description of training was not included in the study conducted by Moosa et al. (2017). In several studies, additional agents were included such as licensed art therapists, psychologists, psychiatrists, Syrian college students, graduate students, and translators.

Three out of the eight reviewed studies reported information related to both elements and met the requirement (Meyer DeMott et al., 2017; Moosa et al., 2017; Feen-Calligan et al., 2020). The studies provided detailed information by stating the questions asked during the intervention and providing enough materials to clearly describe the dosage and content of the intervention. Ugurlu et al. (2016) provided sufficient information regarding what the art therapy session will include and described the time and dosage of intervention with pre and post-assessments. However, the authors did not provide enough information regarding the description of the materials. In the study conducted by Rowe et al. (2017), the researchers provided sufficient information regarding dosage and the process evaluation framework during the intervention phase but there was insufficient information regarding the materials used and the detailed process was missing regarding the art therapy techniques used. Similarly, DroŽdek et al. (2014) provided a table showing the phases of intervention with the content but did not provide enough materials to describe the specificity of the intervention. In the study done by Schouten et al. (2019), the researchers explained the sessions but the specific elements of art therapy were not evident and reported. Therefore, this study did not explicitly describe the intervention procedure and materials used, and the researchers provided only general information. In addition, the study that was conducted by Van Wyk et al. (2012) did not describe materials or provide accessible sources.

Five out of eight studies did not meet this criterion as no implementation fidelity data were collected (Van Wyk et al., 2012; DroŽdek et al., 2014; Ugurlu et al., 2016; Meyer DeMott et al., 2017; Moosa et al., 2017). In article Rowe et al.'s article, the authors described how the therapists were able to follow the planned protocol for administering assessment tools and dose delivered but it did not clearly state the measures used such as observation checklists or self-reports of the implementation by unit analysis. Schouten et al. (2019) applied the protocol checklist that gave therapists ways to direct sessions and the patients reported satisfaction regarding the session. A checklist regarding treatment adherence was included in this study. However, the questionnaire developed by the author was not clearly described and explained thoroughly. In the article Feen-Calligan et al. (2020), the study did not include measures or checklists to determine fidelity implementation but the study provided information regarding analyzing sessions by the team and refining sessions by adding recommendations and tracking changes to the following session or week. The observation checklists or self-reports in addition to the dosage were not present in the study.

None of the reviewed studies met all nine elements of the internal validity criterion. In the group design study conducted by Meyer DeMott et al. (2017), the researchers reported information regarding the assignment of participants to groups but did not clarify if the control group had limited access to intervention. Moreover, in the same study, the differential attrition was less than 10 percent, but the overall attrition was higher than 30 percent. On the other hand, in the study conducted by Feen-Calligan et al. (2020), the independent variable was controlled, control group conditions were described, and the study reported that the control group had no access to intervention. The study carried out by Feen-Calligan et al. (2020) clearly described how participants were assigned to either the treatment or the control group. In their study, Rowe et al. (2017) reported that participants had access to other mental health resources, which is a threat to internal validity. In four of the studies (i.e., 50%), researchers did not indicate whether participants in the baseline condition had access to any of the intervention components (Van Wyk et al., 2012; Ugurlu et al., 2016; Moosa et al., 2017; Schouten et al., 2019). This may have presented a potential threat to the internal validity of the mentioned studies.

All studies that measured socially important outcomes provided clear operational definitions of the dependent variables and reported the effects of the intervention. The only study that met all elements related to the dependent variable(s) was the study conducted by DroŽdek et al. (2014) because in this study the tools used were validated across cultural settings and demonstrated good interrater and test-retest reliability. Rowe et al. (2017) reported that validated assessment tools were used in this study and measures of internal consistency among items for each of the scales were strong. Similarly, the measurement tools used in the study done by Schouten et al. (2019) and the study done by Van Wyk et al. (2012) had been validated and the reliability was good. In addition, Moosa et al. (2017) reported in the study that the measurement tools used were reliable with internal reliability higher than 80. Similarly, the study done by Ugurlu et al. (2016) provided evidence of internal reliability and the study done by Feen-Calligan et al. (2020) provided evidence of reliability and validity. The study that provided three data points was the study done by Meyer DeMott et al. (2017) provided an adequate description of the outcomes measures but did not provide information regarding the internal reliability and validity of the tools.

Regarding data analysis, all reviewed studies met the requirements for this quality indicator. Three elements are included in the data analysis criteria. For a group design study, the study is expected to report effect size statistics and data analysis techniques need to be provided to compare the change in the outcome of the groups (Cook et al., 2014). All group comparison design studies employed appropriate statistical analysis methods that align with their research questions and data collection tools. Additionally, all reviewed studies provided clear descriptions of their data analysis techniques.

### Evidence-Based Classification

Upon evaluation of the methodological soundness of the reviewed studies based on CEC quality indicators and evidence-based classification (Cook et al., 2014), we found that the practice of using art therapy for the treatment of PTSD in refugees falls under the “insufficient evidence” category. As mentioned earlier, according to CEC standards, a study can be considered for evidence-based classification only when they employ a group comparison design (e.g., randomized or quasi-experiment) or a single subject design (Cook et al., 2014). The only two studies that were eligible for classification were the ones that employed quasi-experimental designs (Meyer DeMott et al., 2017; Feen-Calligan et al., 2020). Both studies met more than 50% of quality indicators (see Table 1). However, upon examination of individual intervention results reported by the authors of both studies, we found that the art therapy intervention conducted by Feen-Calligan et al. (2020) yielded positive effects on participants' PTSD symptoms (i.e., the difference between groups was statistically significant in favor of the treatment group). On the other hand, the intervention implemented by Meyer DeMott et al. (2017) yielded neutral effects (i.e., the differences between groups were not statistically significant). This finding means that the ratio of methodologically sound studies with positive effects to methodologically sound studies with neutral/mixed-effects was <2:1, and thus places the treatment practice in the “insufficient evidence” classification.

## Discussion

This systematic review is the first to examine art therapy with a focus on refugees with PTSD symptoms from 2010 to 2020. After a systematic review of the literature and multi-step evaluation of studies for quality indicators, the findings revealed that art therapy as a treatment for PTSD in children and youth refugees meets the “insufficient evidence” classification as per CEC standards. The present systematic review found that the majority of art therapy intervention studies with refugees with PTSD employed a pre-test post-test group design with no control group. In some studies, art therapy has been used in the intervention as an independent component while in other studies art therapy has been used as part of expressive, creative, or group therapy. All studies met the quality indicators related to context and participants, and thus we can deduce that the studies described clearly the features of the context and setting studies provided enough information related to participants' demographics as well as a clear description of the status of participants. We also notice variation among results regarding the other quality indicators such as the description of practice, the fidelity of implementation, intervention agents, data analysis, internal validity, and outcome measures. Most of the studies (five out of eight studies) met the criteria related to intervention agents and most of the studies described the role of the therapist, qualifications, and the specific training. When examining the description of the intervention, only three studies met this quality indicator by providing a clear and detailed description of the intervention with sufficient materials to describe it specifically. Studies that did not meet this quality indicator lacked the element that is related to providing sufficient information on materials relevant to the intervention being examined. As for implementation fidelity, none of the articles fully met this quality indicator, and two articles did not even mention any effort to collect fidelity data. It is critical to note that this methodological limitation has the potential to pose a threat to internal reliability.

Furthermore, four studies did not meet the quality indicator when examining internal validity and two studies met half of the elements. Only two studies met four elements out of the six elements, and only one study met all the elements regarding internal validity (Feen-Calligan et al., 2020). When examining the outcome measures/ dependent variables, we also notice variation among results. Only one study met all elements for this quality indicator and most of the studies did not provide a minimum of three data points per phase. As for the criteria related to data analysis, all the studies met CEC standards as data analysis was adequately conducted and reported. In conclusion, the lowest scores across studies were for meeting implementation fidelity (i.e., 21%) and internal validity criteria (i.e., 52%).

### Limitations of the Study

Since the initial search yielded a very limited number of studies on children refugees, studies with participants of all ages were included in this review. Most of the studies located during the search procedure were qualitative case studies and this indicated that few experimental studies were conducted to test the efficacy of an intervention. This may reflect that researchers need to shift toward empirical evidence more than case studies or qualitative research. Second, the search is limited to peer-reviewed journal articles, and thus excluding dissertations or other types of publications may be a limitation. Another limitation, including peer-reviewed journal articles that are in the English language only may have left out findings from important work written in other languages. Consequently, it can be noted that the low number of articles included in this review may limit generalizing of the findings. Moreover, the number of studies located may be insufficient to present a statistical measurement or meta-analysis. Results and findings also show the importance and need for future studies and more experimental studies because it is essential to improve the practices and methods of implementation. Future systematic reviews may also need to improve search procedures by examining more databases or including additional resources to improve search procedures yielding results that are more accurate. It is also essential to examine these practices with other participants (military, participants who witnessed war but did not leave the country, and others) or participants suffering from other types of disorders or symptoms to compare the effectiveness of these practices.

### Implications for Research, Practice, and Policy

The majority of reviewed studies used pre-post group designs with the absence of a control group, and very few articles employed experimental research methodology (e.g., group experiment, quasi-experimental, single-subject design). Knowing that intervention studies that employ single-group pre-post designs are limited by serious threats to internal validity (Shadish, 2002), future research should focus on experimental methodology to investigate the effectiveness of art therapy on PTSD symptoms in refugees. We also suggest that more experimental studies are warranted across other different contexts to identify how art therapy approaches can be implemented effectively. Therefore, this field or area of practice needs to be further explored, developed, and enhanced since art therapy-based interventions may lead to positive effects on individuals suffering from PTSD, as shown in one methodologically sound study among the reviewed literature (Feen-Calligan et al., 2020). Further systematic reviews that combine both qualitative and quantitative methodologies may be included to explore the elements that may affect and decrease traumatic symptoms. This review developed a bridge between what literature presents, what therapists know, and therapists' practice. To improve the transferability of practice, further quantitative studies are needed that aim at enhancing implementation fidelity and having sufficient information regarding internal validity. Future studies need to provide more detailed materials or resources when describing the practice in the studies. After examining the table and comparing the ages of participants, it is essential to improve practices in future studies by focusing on children as participants since most of the studies included adolescents or adults. Other factors may also have an impact on the effectiveness of the intervention and these variables should be taken into consideration when interpreting data. It may also be difficult to reach conclusions about the most effective interventions and practices because of the small sample groups. It would be important to conduct further comparative studies among different contexts and wider populations to draw firmer conclusions about the most effective interventions for traumatized children and adolescent refugees.

## Data Availability Statement

The original contributions presented in the study are included in the article/supplementary material, further inquiries can be directed to the corresponding author/s.

## Author Contributions

All authors listed have made a substantial, direct, and intellectual contribution to the work and approved it for publication.

## Conflict of Interest

The authors declare that the research was conducted in the absence of any commercial or financial relationships that could be construed as a potential conflict of interest.

## Publisher's Note

All claims expressed in this article are solely those of the authors and do not necessarily represent those of their affiliated organizations, or those of the publisher, the editors and the reviewers. Any product that may be evaluated in this article, or claim that may be made by its manufacturer, is not guaranteed or endorsed by the publisher.
